# Safety and clinical efficacy of sintilimab (anti-PD-1) in pediatric patients with advanced or recurrent malignancies in a phase I study

**DOI:** 10.1038/s41392-023-01636-9

**Published:** 2023-10-13

**Authors:** Yi Que, Juan Wang, Feifei Sun, Shan Wang, Jia Zhu, Junting Huang, Zhenzhen Zhao, Li Zhang, Juan Liu, Jiaqian Xu, Zijun Zhen, Xiaofei Sun, Suying Lu, Yizhuo Zhang

**Affiliations:** 1 Department of Pediatric Oncology, Sun Yat-sen University Cancer Center, State Key Laboratory of Oncology in South China, Collaborative Innovation Center for Cancer Medicine, Guangzhou, 510060 PR China; 2 Department of Surgical Oncology, National Clinical Research Center for ChildHealth and Disorders, Ministry of Education Key Laboratory of Child Development and Disorders, China International Science and Technology Cooperation Base of Child Development and Critical Disorders, Chongqing Key Laboratory of Pediatrics, Children’s Hospital of Chongqing Medical University, Chongqing, 400014 China

**Keywords:** Paediatric cancer, Immunotherapy

## Abstract

The aim of this phase I study is to evaluate, for the first time, the safety and efficacy of sintilimab in pediatric patients diagnosed with advanced or recurrent malignancies. During the dose escalation phase, patients received a single intravenous infusion of sintilimab at varying doses of 1, 3, and 10 mg/kg. The primary endpoints included the identification of dose-limiting toxicities (DLTs) as well as the evaluation of safety and tolerance. Secondary endpoints focused on assessing objective response rate (ORR), progression-free survival (PFS), and overall survival (OS). A total of 29 patients were enrolled, including 10 individuals diagnosed with Hodgkin lymphoma (HL) and 19 patients with various other tumor categories. Notably, diverse pathological types such as thymoma, choroid plexus carcinoma, and NK/T-cell lymphoma were also included in the study cohort. By the safety data cutoff, most adverse events were grade 1 or 2, with grade 3 or higher treatment-related adverse events (TRAE) occurring in 10% of patients. Among the 27 evaluated subjects, four achieved confirmed complete response (CR) while seven patients exhibited confirmed partial response (PR). Additionally, seven patients maintained disease (SD) during the study period. Notably, sintilimab demonstrated remarkable tolerability without DLTs and exhibited promising anti-tumor effects in pediatric HL. Whole-exome sequencing (WES) was conducted in 15 patients to assess the mutational landscape and copy number variation (CNV) status. The completion of this phase I study establishes the foundation for potential combination regimens involving sintilimab in childhood cancer treatment. The trial is registered on ClinicalTrials.gov with the identifier NCT04400851.

## Introduction

In the past few years, immune checkpoint inhibitors (ICIs), particularly programmed cell death protein 1 (PD-1) or programmed cell death protein ligand 1 (PD-L1), have brought advancements to adult cancer treatment, resulting in remarkable breakthroughs.^1–3^ It is worth noting that blockade of PD-1/PD-L1 pathway have demonstrated significant improvements in the outcomes of adult cancer patients. However, research on checkpoint inhibition, specifically targeting PD-1/PD-L1 in pediatric patients, has been limited.^4^ Studies such as KEYNOTE-051 have shown the effectiveness of pembrolizumab in relapsed or refractory HL and certain rare tumor types, such as mesothelioma and adrenocortical carcinoma.^5^ Similarly, nivolumab has exhibited safety and anti-tumor activity in pediatric patients with relapsed or refractory non-central nervous system solid tumors or lymphoma.^6^ Atezolizumab, a PD-L1 inhibitor, was well-tolerated with generally comparable exposure in pediatric populations, although only four patients (5%) achieved partial objective responses.^7^ Despite existing reports on PD-1 in pediatric patients, there is a significant dearth of clinical trials involving domestically produced PD-1 antibodies. Furthermore, although a series of progress has been made in the study of potential mechanisms of pediatric cancer in recent years, the standard treatment approach for pediatric cancer patients still consists of surgery, chemotherapy, and radiation therapy.^8–10^ Unfortunately, the prognosis for relapsed or advanced solid tumors in children remains unfavorable.^11,12^ Acknowledgement of the low survival rate of recurrent or advanced pediatric cancer patients^13^ promote the research and development of immunotherapy treatments, which motivates us to investigate its potential for improving the survival of pediatric cancer patients.

Currently, there is limited data regarding the effect of PD-1 antibodies in China, especially in pediatric populations. Prospective clinical trials have been conducted to explore the potential of inhibitors like pembrolizumab and nivolumab in the treatment of pediatric patients,^5,6^ the majority of which are Caucasians with a small proportion of Asian participants. Nevertheless, there exists a substantial potential demand for PD-1 antibodies among pediatric cancer patients. We have conducted a retrospective exploration of the safety and anti-tumor activity of PD-1 antibody monotherapy, as well as its combination with other regimens, in pediatric cancer patients of Asian descent,^14^ supporting the practical foundations for PD-1 inhibitors for advanced pediatric cancers in a prospective study.

Sintilimab, characterized as a fully humanized immunoglobulin G4 anti-PD-1 monoclonal antibody, has demonstrated promising anti-tumor activity and a favorable safety profile across various malignancies. Notably, it has demonstrated significant clinical advancements in NK/T-cell lymphoma. In the ORIENT-4 trial, Tao et al. found that sintilimab exhibited effectiveness and favorable tolerability in patients with relapsed/refractory extranodal natural killer/T-cell lymphoma (r/r ENKTL), suggesting its potential as an innovative therapeutic strategy for managing ENKTL.^15^ In the ORIENT-1 study, Shi et al. revealed that among the full analysis set (*n* = 92), 74 patients (80.4%: 95% CI 70.9-88.0) achieved an objective response indicating the potential efficacy of sintilimab in treating relapsed or refractory classical HL.^16^ Li et al. observed that the incorporation of sintilimab into the treatment regimen resulted in extended PFS compared to conventional chemotherapy in locally advanced head and neck squamous cell carcinoma (HNSCC), all while maintaining a comparable safety profile.^17^ Moreover, in a multicenter, randomized, double-blind phase 3 trial, the use of sintilimab in combination with cisplatin and paclitaxel as a first-line treatment demonstrated substantial advantages in both OS and PFS for patients diagnosed with advanced or metastatic esophageal squamous cell carcinoma.^18^ In 2018, the National Medical Products Administration of China granted accelerated approval for sintilimab for the treatment of classical HL, and in the same year, the US FDA accepted an Investigational New Drug application for sintilimab.^19^ It is important to know that the therapeutic potential of sintilimab extends beyond the specified tumor types, warranting further exploration across a wider range of cancers. However, its safety and efficacy have not been evaluated in pediatric cancer. The acceptable safety profile of sintilimab serves to alleviate concerns related to severe TRAE in patients with pediatric cancer.

It is crucial to emphasize the significance of domestically produced PD-1 antibodies in meeting the requirements of pediatric patients. In this article, we conducted a phase I study of sintilimab in children with recurrent/advanced cancer, including 10 HL, 3 mesenchymal malignant tumors, 2 anaplastic ependymomas, and some unique pathological types. The genomic landscape of patients treated with sintilimab was also evaluated. This in-depth exploration provides valuable information about the underlying biology and potential biomarkers associated with treatment response. Previous research has not extensively investigated the use of sintilimab in this particular patient population. To our knowledge, this represents the initial prospective clinical investigation of sintilimab in pediatric patients diagnosed with advanced malignancies. Sintilimab exhibited a satisfactory overall safety profile without encountering any DLTs, and objective clinical responses were observed at each administered dose level. Our study provides unique insights into the potential of sintilimab as a treatment option for pediatric cancer and lays a foundation for future combined PD-1 antibody applications.

## Results

### Patients and treatments

**Fig. 1:**
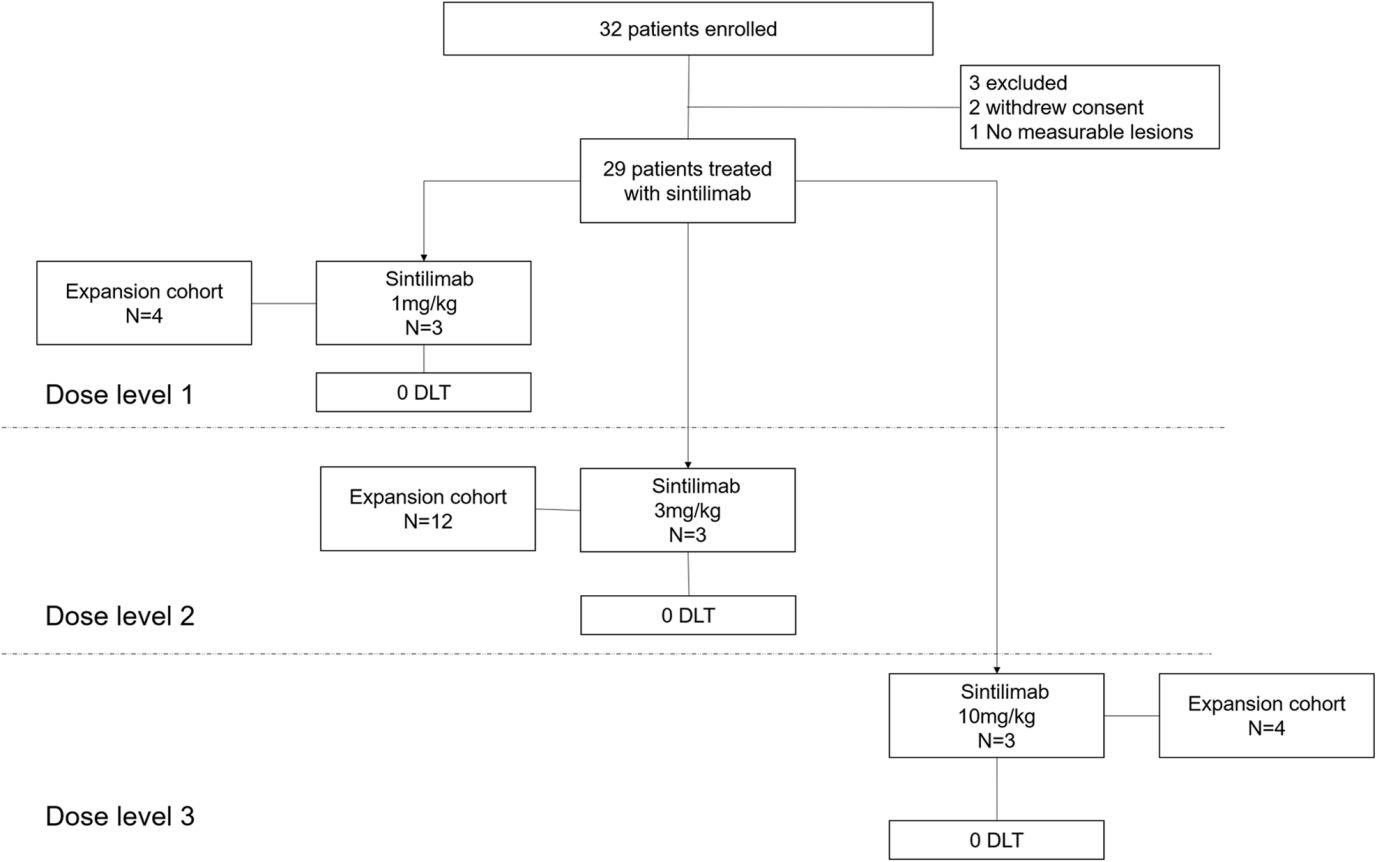
CONSORT diagram of the study. Out of the 32 patients initially screened, 29 were enrolled to receive sintilimab treatment. The study utilized the standard 3 + 3 design. DLT represents dose-limiting toxicity

From December 2020 through July 2022, 29 subjects were treated with sintilimab in three dose-escalating cohorts of 1 mg/kg (*n* = 7), 3 mg/kg (*n* = 15) and 10 mg/kg (*n* = 7) (Fig. 1). Part A involved 9 patients in total for dose escalation, with 3 patients assigned to each of the 1 mg/kg, 3 mg/kg, and 10 mg/kg dosing groups. During Part B, the dose expansion phase included a cohort of 20 patients, with 4 patients each in the 1 mg/kg and 10 mg/kg groups, and 12 patients in the 3 mg/kg group. The subjects had the following diagnoses: HL (*n* = 10), diffuse large B-cell lymphoma (*n* = 1), primary mediastinal large B-cell lymphoma (*n* = 1), NK/T-cell lymphoma (*n* = 1), malignant mesothelioma (*n* = 1), mesenchymal malignant tumor (*n* = 3), anaplastic ependymoma (*n* = 2), neuroblastoma (*n* = 1), melanoma (*n* = 1), epithelioid sarcoma (*n* = 2), Ewing’s sarcoma (*n* = 1), undifferentiated sarcoma (*n* = 1), thymoma (*n* = 1), yolk sac tumor (*n* = 1), germinoma (*n* = 1), and atypical choroid plexus papilloma (*n* = 1). The median follow-up was 47.27 weeks (IQR 10.57–122.14). The median age of the subjects were 10 years, with a total of 20 male and 9 female subjects. The baseline median body weight was 30.0 kg. Among the enrolled subjects, two (6.9%) subjects had an Eastern Cooperative Oncology Group (ECOG) performance score of 1, while 27 (93.1%) subjects had an ECOG score of 0. Participants had received extensive prior treatment, including a median of 2 prior lines of systemic therapy (range: 0-8). All subjects had a history of previous systemic chemotherapy (Table 1**)**.

**Table 1:** Baseline characteristics of patients

Characteristics n (%)	All patients (*n* = 29)
Age, years	
1-10	13
10-18	16
Sex	
Male	20
Female	9
Body weight (kg)	
<30	14
≥30 and <50	9
≥50	6
Diagnosis	
Hodgkin lymphoma	10
Diffuse large B-cell lymphoma	1
Primary mediastinal large B-cell lymphoma	1
NK/T-cell lymphoma	1
Malignant mesothelioma	1
Mesenchymal malignant tumor	3
Anaplastic ependymoma	2
Neuroblastoma	1
Melanoma	1
Epithelioid sarcoma	2
Ewing’s sarcoma	1
Undifferentiated sarcoma	1
Thymoma	1
Yolk sac tumor	1
Germinoma	1
Atypical choroid plexus papilloma	1
Previous lines of chemotherapy	
1	9
2	7
≥3	13
ECOG performance status	
0	27
1	2

### Treatment-related toxicity

Up until the cutoff date of December 15, 2022, the patients in the study received a median of 5 cycles of sintilimab treatment (range 1-24). The administration of sintilimab was generally well tolerated, as indicated by a total of 89.7% (26 out of 29) of patients experiencing at least one TRAE. The most frequently observed TRAEs are provided in Table 2, including 10 cases of anemia (34.5%), 2 cases of fatigue (6.9%), 5 cases of hypothyroidism (17.2%), 4 cases of hyperthyroidism (13.8%), 1 case of nausea (3.4%), 4 cases of decreased white blood cell count (13.8%), 4 cases of pruritus (13.8%), 3 cases of decreased neutrophil count (10.3%), 11 cases of pyrexia (37.9%), 3 cases of cough (10.3%), 2 cases of myalgia (6.9%), 3 cases of headache (10.3%), and 3 (10.3%) patients who experienced grade 3 or higher TRAEs, including one case each of increased aspartate aminotransferase (Grade 3), increased alanine aminotransferase (Grade 3) and myositis (Grade 3), as well as 2 cases of decreased platelet count (Grade 3). Sintilimab did not show a dose (1-10 mg/kg)-dependent safety profile. Treatment-related severe adverse events (SAEs) did not occur. Two TRAEs resulted in discontinuation of the treatment due to myositis or immune pneumonia. No severe infusion reactions were reported during the study. We also conducted separate analyses for adverse side effects in hematological tumors and solid tumors, as presented in Supplementary Table S1. Immune-related adverse events (irAEs) were primarily observed at grades 1-2 and included infusion reactions (7%), hypothyroidism (7%), hyperthyroidism (7%), rash (7%), and pneumonitis (3%) (Table 3).

**Table 2:** Summary of sintilimab safety profile

Dose	1 mg/kg (*n* = 7)	3 mg/kg (*n* = 15)	10 mg/kg (*n* = 7)	1 mg/kg (*n* = 7)	3 mg/kg (*n* = 15)	10 mg/kg (*n* = 7)
Grade	Grade1-2	Grade1-2	Grade1-2	Grade 3-4	Grade 3-4	Grade 3-4
Total number of patients	6(85.7%)	14(93.3%)	6(85.7%)	2(28.6%)	1 (6.7%)	0
Anemia	3(42.9%)	5(33.3%)	2(28.6%)	0	0	0
Fatigue	1(14.3%)	1(6.7%)	0	0	0	0
Pyrexia	3(42.9%)	6(40.0%)	2(28.6%)	0	0	0
Aspartate aminotransferase increased	0	2(13.3%)	0	1(14.3%)	0	0
Alanine aminotransferase increased	0	2(13.3%)	0	1(14.3%)	0	0
Hypothyroidism	1(14.3%)	2(13.3%)	2(28.6%)	0	0	0
Hyperthyroidism	1(14.3%)	3(20.0%)	0	0	0	0
Nausea	0	1(6.7%)	0	0	0	0
Rash, maculopapular	1(14.3%)	2(13.3%)	0	0	0	0
Diarrhoea	0	1(6.7%)	0	0	0	0
Abdominal pain	0	2(13.3%)	1(14.3%)	0	0	0
Decreased white blood cell count	0	2(13.3%)	2(28.6%)	0	0	0
Decreased appetite	0	2(13.3%)	0	0	0	0
Pruritus	1(14.3%)	3(20%)	0	0	0	0
Decreased platelet count	1(14.3%)	0	1(14.3%)	1(14.3%)	1 (6.7%)	0
Arthralgia	0	1(6.7%)	0	0	0	0
Decreased neutrophil count	0	2(13.3%)	1(14.3%)	0	0	0
Pneumonitis	0	1(6.7%)	0	0	0	0
Hypoalbuminemia	1(14.3%)	0	1(14.3%)	0	0	0
Hypokalaemia	0	1(6.7%)	0	0	0	0
Hypocalcaemia	0	0	1(14.3%)	0	0	0
Cough	2(28.6%)	1(6.7%)	0	0	0	0
Myalgia	1(14.3%)	1(6.7%)	0	0	0	0
Headache	1(14.3%)	2(13.3%)	0	0	0	0
Vomitting	0	1(6.7%)	1(14.3%)	0	0	0
Chest tightness	1(14.3%)	1(6.7%)	0	0	0	0
Constipation	0	1(6.7%)	1(14.3%)	0	0	0
Myositis	0	0	0	1(14.3%)	0	0

**Table 3:** Immune-related adverse effects and infusion reactions

	Grade 1-2	Grade 3-4
Infusion reactions	2 (7%)	0
Hypothyroidism	2 (7%)	0
Hyperthyroidism	2 (7%)	0
Myositis	0	1 (3%)
Pneumonitis	1 (3%)	0
Aspartate aminotransferase increased	0	1 (3%)
Alanine aminotransferase increased	0	1 (3%)
Rash, maculopapular	2 (7%)	0

In one case, a 14-year-old boy diagnosed with primary mediastinal large B-cell lymphoma experienced disease progression after completing six cycles of chemotherapy using the DA-R-EPOCH regimen. Subsequently, he received sintilimab treatment, and the best response obtained was SD, with the maximum diameter of the tumor reduced by 19%. However, after 5 courses of treatment, the patient developed a grade 1 adverse effect of immune pneumonia and voluntarily withdrew from the treatment (Supplementary Fig. S1).

### Treatment responses

**Fig. 2:**
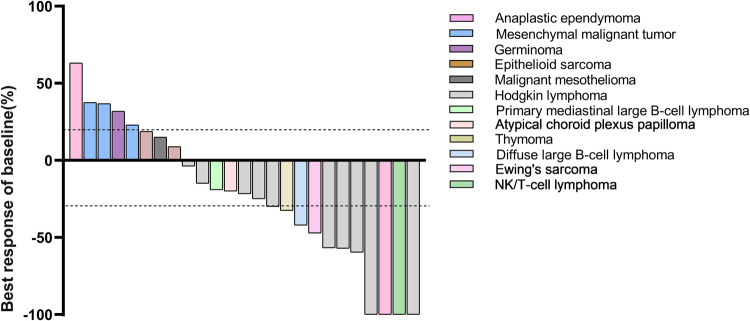
Anti-tumor activity of sintilimab according to RECIST version 1.1. The waterfall plot shows the best percentage reduction in tumor burden from baseline. Included patients were those with relapsed or refractory HL (10 patients) or any tumor type except HL (15 patients). Each bar represents an individual patient. Dashed lines at a 20% increase and a 30% decrease serve as the thresholds for defining PD and PR, respectively, in accordance with RECIST 1.1 criteria

Among 17 patients with other types of tumors, referred to as “except-HL” tumors, 2 CR (NK/T lymphoma and anaplastic ependymoma), 3 PR (1 diffuse large B-cell lymphoma, 1 Ewing’s sarcoma, and 1 thymoma) and 3 SD (1 epithelioid sarcoma, 1 atypical choroid plexus papilloma, and 1 primary mediastinal large B-cell lymphoma) were observed, as evaluated by the investigator following RECIST v1.1 criteria (Fig. 2, Supplementary Fig. S2). In other types of tumors, the ORR was 29.4%, and the disease control rate (DCR) was 47.1%. In the group of patients with lymphoma, the ORR was 61.5% (8 out of 13), and the DCR was 100% (13 out of 13), with one NK/T lymphoma patient and one diffuse large B-cell lymphoma patient experiencing CR and PR, respectively. Among 10 HL patients, the overall ORR and DCR were 60.0% (6/10) and 100%, respectively, according to IWG 2007 criteria (Table 4). Two of 10 (20%) HL patients experienced CR.

**Table 4:** Antitumor activity

Histology	Hodgkin lymphoma (*n* = 10)	Other types of tumors (*n* = 17)	Total (*n* = 27)	ORR, DCR
1 mg/kg (*n* = 6)	1CR, 1PR	1CR, 1PR, 2PD	2CR, 2PR, 2PD	66.7%, 66.7%
3 mg/kg (*n* = 14)	1CR, 2PR, 4 SD	1CR, 1 SD, 5PD	2CR, 2PR,5 SD, 5PD	28.6%, 64.3%
10 mg/kg (*n* = 7)	1PR	2PR, 2 SD, 2PD	3PR,2 SD, 2PD	42.9%, 71.4%
Total (*n* = 27)	2CR, 4PR, 4 SD	2CR, 3PR, 3 SD, 9PD	4CR, 7PR,7 SD,9PD	40.7%, 66.7%
ORR, DCR	60.0%, 100%	29.4%, 47.1%	40.7%, 66.7%	/

In a specific case, a 14-year-old boy was diagnosed with thymoma and myasthenia gravis. The tumor did not respond after receiving multiple chemotherapies (cyclophosphamide, cisplatin, and pirarubicin). WES revealed high expression of PD-L1 with a tumor proportion score (TPS) score of 98%. Consequently, he was enrolled to receive sintilimab treatment. After one cycle of treatment, the patient developed grade 3 immune hepatitis and discontinued anti-PD-1 therapy. The patient experienced pseudoprogression, as the tumor initially showed progression, but later the efficacy was evaluated as partial remission on computed tomography findings (Supplementary Fig. S3).

**Fig. 3:**
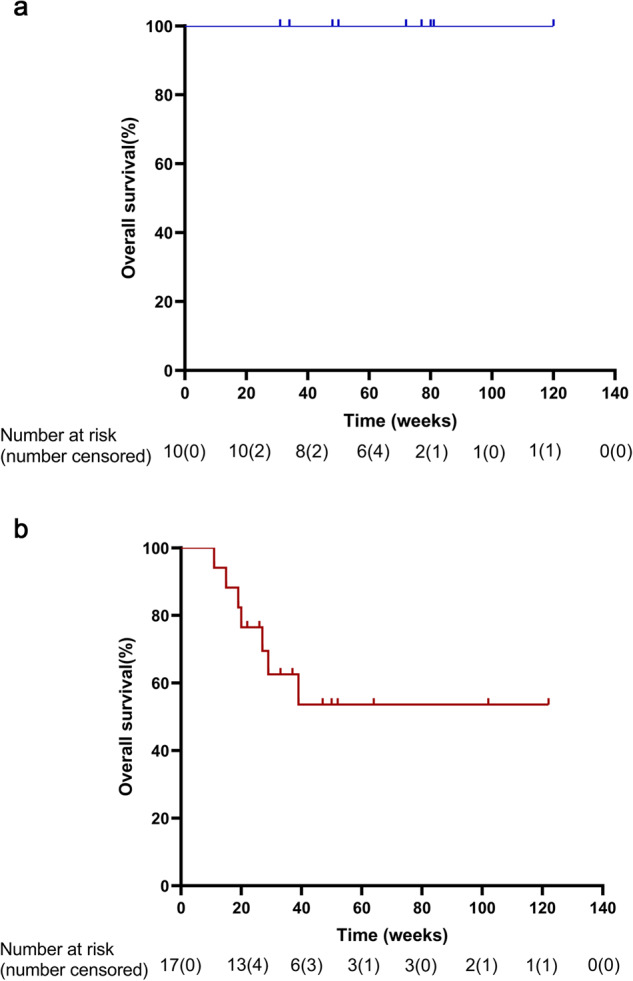
Kaplan‒Meier analysis of overall survival estimates in patients with relapsed or refractory HL (**a**) and any other tumor type except HL (**b**)

**Fig. 4:**
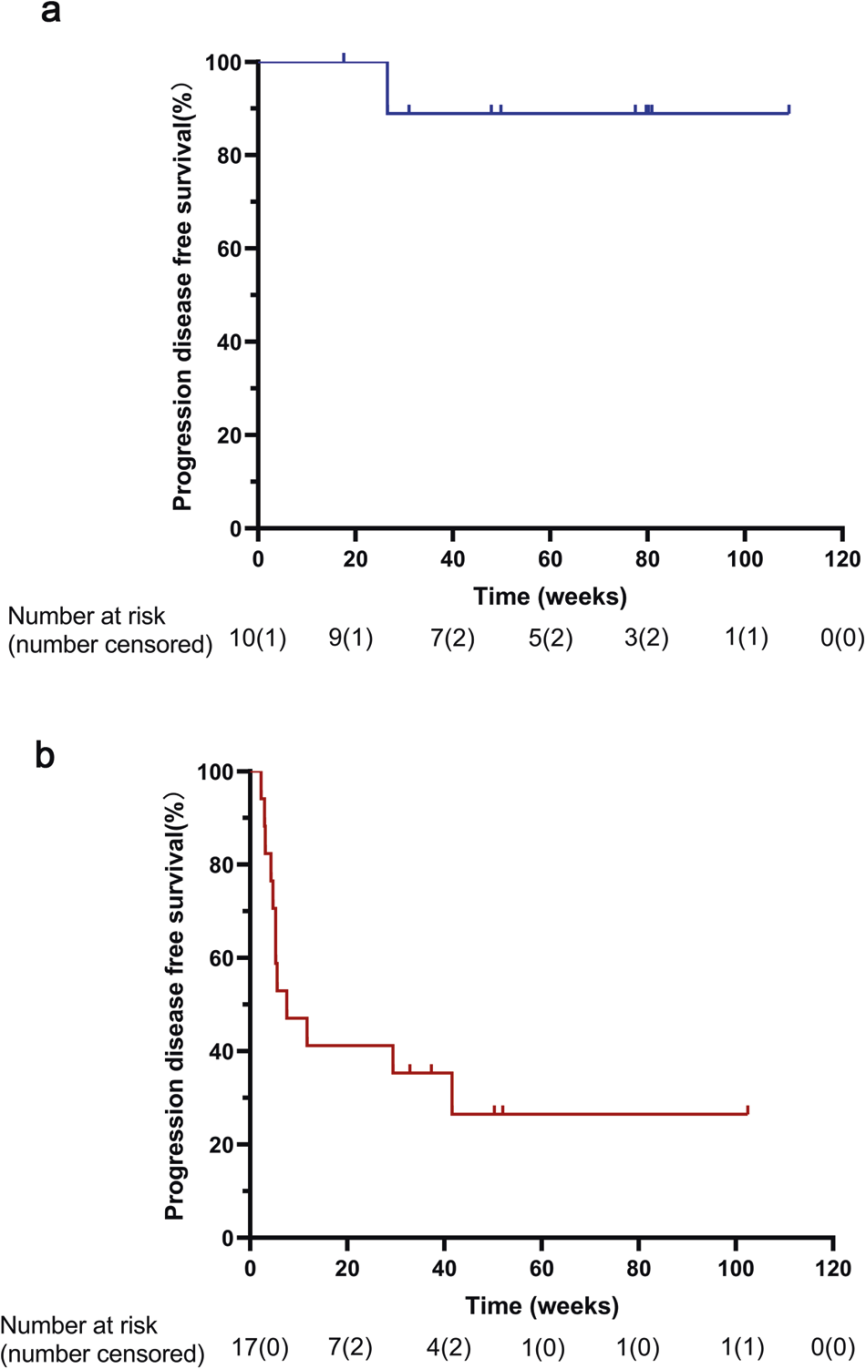
Kaplan‒Meier estimates of disease progression survival in patients with relapsed or refractory HL (**a**) and any other tumor type except HL (**b**)

By the cutoff date, 24 patients had discontinued their treatment. Among this group, there were seven cases of patient mortality during the follow-up period, and none of these were attributed to the intervention administered in the study. Three patients remained in the study. Three CR patients (2 HL and 1 NK/T lymphoma) voluntarily discontinued treatment after maintaining CR for more than three months. Two HL patients, with 1 PR and 1 SD patient, discontinued treatment after two cycles of sintilimab due to the decision to combine it with chemotherapy. One HL patient with PR discontinued treatment to prepare for hematopoietic stem cell transplantation. Among all 27 patients, the median PFS was not reached. The median OS was not reached for the overall population, HL patients, or patients with other types of tumors except HL (Fig. 3). The median PFS was 7.57 weeks for 17 other types of tumors, and it was not reached for the 10 HL patients (Fig. 4).

### Analysis of biomarkers and subgroups to determine their correlation with clinical efficacy

**Fig. 5:**
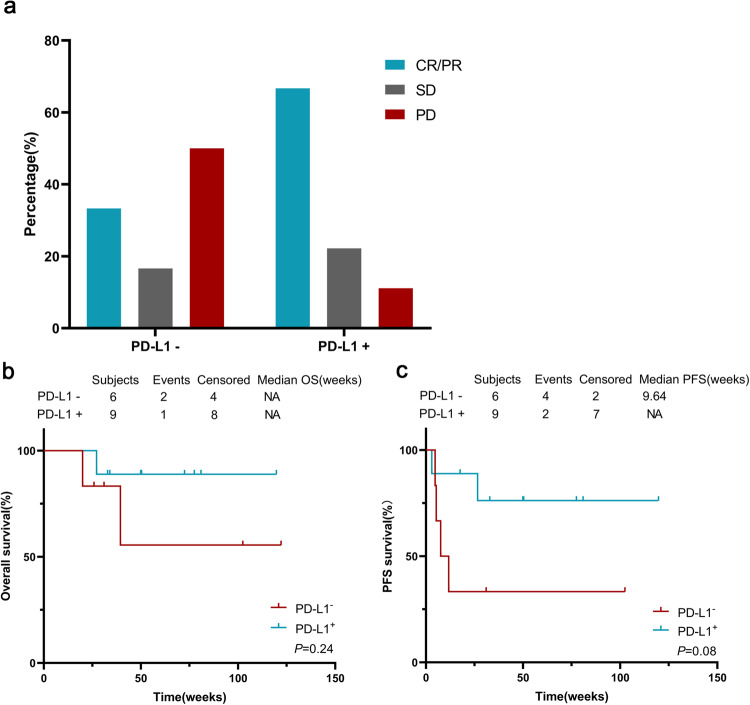
Evaluating the relationship between clinical efficacy and PD-L1 expression as determined by IHC staining in tumor biopsies. Subdivisions of PD-L1 expression by anti-PD-L1 antibody (DACO, 22C3) IHC staining to determine their correlation with objective response (**a**), OS (**b**) and PFS (**c**). PD-L1+ is defined as TPS ≥ 1% and CPS ≥ 1%, while PD-L1- is defined as TPS < 1% and CPS < 1%. The percentages of surviving patients are indicated at the specified time points. The numbers of patients at risk at the indicated time points are shown below the x-axis

Patients exhibiting PD-L1-positive expression (with a TPS ≥ 1%) derived greater benefits from sintilimab treatment compared to individuals with PD-L1-negative status (with a TPS < 1%), although the difference was statistically insignificant (66.7% ORR versus 33.3% ORR, *p* = 0.314) (Fig. 5a). Furthermore, PD-L1-positive subjects showed a numerically better OS (HR = 0.242 (95% CI 0.0223–2.625), *p* = 0.243) (Fig. 5b) and PFS (HR = 0.226 (95% CI 0.041–1.246), *p* = 0.087) (Fig. 5c) from sintilimab treatment than PD-L1-negative subjects, although the difference was not statistically significant. Additionally, CD8-positive subjects achieved a higher DCR (81.8% versus 50%) and ORR (54.5% versus 50%) after sintilimab treatment compared to CD8-negative subjects, but the difference was not statistically significant (*p* > 0.05) (Supplementary Fig. S4).

### Genomic profiling and gene panel measurement of patients treated with sintilimab

**Fig. 6:**
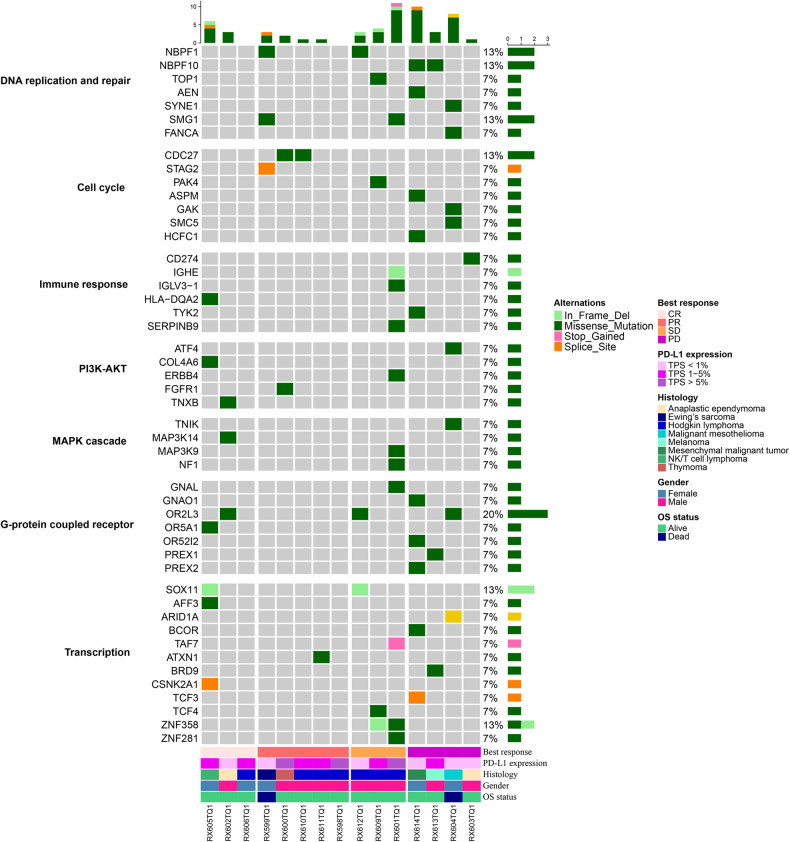
Genomic profiling was conducted using WES on both FFPE tumor and matched peripheral blood samples obtained from 15 eligible subjects

In this study, we conducted WES of 15 patients to comprehensively characterize the mutational landscape of advanced and refractory pediatric patients. Our genomic analyses revealed that certain genetic alterations in important pathways occurred more frequently in the nonresponse group (SD/PD) patients compared to the response group (CR/PR) patients. G-protein-coupled receptors (GPCRs), constituting the largest gene family of cell membrane-associated molecules, actively engaged in signal transmission, have been linked to therapy resistance and immune evasion.^20^ Consistent with existing literature, the results indicated that nonresponse to ICIs was associated with activation of the GPCR pathway. The mutation rates of GNAL, GNAO1, OR2L3, OR52I2, PREX1, and PREX2 were higher in the PD/SD group compared to the CR/PR group. The nonresponse group also exhibited a higher number of mutated genes in the DNA replication and repair pathway. Specifically, CR patients had no mutations in the related genes of this pathway, while the mutation rate of NBPF10 was 33.3% in the response group compared to 0% in the nonresponse groups (Fig. 6).

Previous investigations have reported that both the PI3K and MAPK pathways are involved in PD-1/PD-L1 regulation.^21,22^ An HL patient who achieved SD as the best response and progressed after 17.57 weeks exhibited a higher frequency of mutated genes related to the PI3K and MAPK pathways, including ERBB4, MAP3K9 and NF1, compared to HL patients who respond well to ICIs.

Our study revealed differences in copy number losses and gains between the CR/PR group and the PD/SD group. Copy number losses of PDGFA and CDK15 and copy number gains of ABCD2 were observed in the nonresponse group, whereas the CR/PR group had copy number losses of E2F1 and GGT7 and copy number gains of PLEKHA3 and TRIM21. Further investigation is needed to clarify the functional outcomes associated with these CNVs related to the response to ICIs **(**Supplementary Fig. S5**)**.

We further analyzed the microsatellite stability status of these patients. Supplementary Fig. S6 summarizes alterations in the main mismatch repair (MMR) genes MLH, PMS2, MSH2, MSH6, EPCAM, POLE and POLD1. All pediatric patients analyzed exhibited microsatellite stable (MSS), with some CNVs but no single nucleotide polymorphisms (SNPs) in MMR genes. MSH2 and MSH6 amplifications/gains were identified in 66.7% (2/3) and 100% (3/3) of patients, respectively, in the PD-L1 (TPS > 5%) group. In the PD-L1 (TPS < 1%) group, amplifications/gains of MSH2 and MSH6 was identified in 33.3% (2/6) for each gene. In the PD-L1 (TPS 1-5%) group, amplifications/gains of MSH2 and MSH6 was identified in 50% (3/6) for each gene. The patient with thymoma exhibited amplification/gain of all MMR genes.

In a separate case, a 5-year-old girl was diagnosed with choroid plexus papillary carcinoma. The disease progressed after multiple lines of chemotherapy. Subsequently, high-throughput sequencing of cancer gene mutations (295 genes related to cancer pathogenesis and targeted therapy) was performed. Mutations in TP53, ROS1, FANCD2, NOTCH1, and MEF2B were identified, with a tumor mutational burden (TMB) of 8.16 mut/Mb. The patient met the inclusion criteria and received treatment with sintilimab, resulting in disease control with a median PFS of 39 weeks.

Peripheral blood samples were obtained from patients receiving sintilimab before and after 2 treatment cycles. Quantification of nine cytokines was performed. The results demonstrated that after 2 cycles of anti-PD-1 treatment, the levels of IFN-γ, TNF-α and IL-4 were significantly upregulated, while the level of IL-9 was downregulated. The levels of other cytokines and the ratios of IL-1b, IL-7, IP-10, MIP-1b, and RANTES remained similar in pre- and post-treatment plasma samples **(**Supplementary Fig. S7**)**.

## Discussion

During this phase I trial, it was observed that sintilimab demonstrated good tolerability in children, with no DLTs reported, even at the highest dose of 10 mg/kg. The frequency and nature of adverse events associated with this drug were in line with what has been documented in adults.^18,23,24^ Sintilimab, a humanized IgG4 anti-PD1 monoclonal antibody, has a wide safety range (1-10 mg/kg), and is primarily eliminated through catabolism. Various intrinsic factors affecting pharmacokinetics (PK) have been assessed in adults, including race, tumor type, age, creatinine clearance (37.1 to 272.2 mL/min), mild hepatic impairment, ECOG performance status, antidrug antibody (ADA), body weight, sex, and baseline albumin. These factors were found to have no clinical meaningful effect on the PK of sintilimab (https://www.fda.gov/media/156022/download; https://img.innoventbio.com/ppdf/new1.pdf). Pembrolizumab and Nivolumab, which are also humanized IgG4 anti-PD1 monoclonal antibodies, share the same elimination pathway as sintilimab. They have cross-validated the expected results, demonstrating similar clinical meaningful exposure when administrated intravenously using body weight-based dosing in both pediatric and adult patients (https://www.accessdata.fda.gov/drugsatfda_docs/label/2023/125514s136lbl.pdf; https://www.accessdata.fda.gov/drugsatfda_docs/label/2023/125554s119lbl.pdf). Considering the above information, it can be anticipated that similar clinically meaningful exposure will be achieved when sintilimab is administrated intravenously using the same body weight-based dosing in pediatric and adult patients.

Sintilimab showed a flat exposure-safety/efficacy relationship in adult patients. Our study found that sintilimab did not exhibit a dose-dependent safety profile within the range of 1-10 mg/kg. This suggests that there are no differential exposure/dose- dependent response relationships between adult and pediatric patients or between sintilimab and other anti-PD-1 drugs.

Considering the anticipated similar exposure between adult and pediatric patients, the observed near-saturated levels of PD-1 receptor occupancy across a various dose levels (1 to 10 mg/kg), the flat exposure/dose-safety relationship in adult and pediatric patients and the absence of clinically meaningful effects on sintilimab PK in adult patients with bodyweights ranging from 36-124.4 kg when administrated at a dose of 200 mg Q3W, the 3 mg/kg Q3W dose regimen is likely positioned within the plateauing region of the exposure-response curves (https://www.fda.gov/media/156022/download). Therefore, a dose of 3 mg/kg with a maximal dose of 200 mg was selected as the recommended phase 2 dose (RP2D).

We observed a lower frequency of grade 3 or grade 4 hematological and non-hematological toxicity in our study compared to that reported in adults, indicating better tolerance in children. Notably, one patient with thymomas experienced irAEs, including myositis and grade 3 hepatitis. Supportive care and corticosteroids were administered to the patient, resulting in symptomatic improvement in the effusion. This finding is consistent with previous literature on the safety of PD-1 in patients with thymomas. In a phase I trial, the administration of avelumab to seven patients with thymoma and one patient with thymic carcinoma resulted in the occurrence of myositis and grade 3-4 irAEs in 38% and 25% of patients, respectively. As a result of these toxicities, five patients had to discontinue the treatment.^25^ A total of 41 patients with thymic carcinoma received pembrolizumab, and among them, six patients experienced grade 4 irAEs. However, there were positive treatment outcomes observed, with 20% of patients achieving a PR and 53% achieving SD.^26^ Although the use of ICIs in thymoma patients holds promise, it is important to note that it is also associated with high rates of toxicity. The adverse events observed can be ascribed to the inherent capacity of thymic malignancies to trigger autoimmunity.

Among the 29 patients treated, two patients in the cohort discontinued the study before their initial scheduled imaging evaluation. Among the 27 patients evaluated for response, two were noted to have disease progressed in an external hospital and withdrew from the study, making it impossible to determine the specific tumor size for these two individuals. Patients with lymphoma showed clinical benefit with sintilimab. Of the ten patients with HL, six achieved an objective best response (2 CR, 4 PR), and four showed SD, with a response rate similar to that reported in adults with HL. The remaining three patients with lymphoma achieved disease control (1 patient with NK/T lymphoma achieved CR, 1 patient with a diffuse large B-cell lymphoma achieved PR, and 1 patient with a primary mediastinal large B-cell lymphoma had SD). Amplification of PD-L1 is thought to play a role in the elevated response rate observed with ICIs.^27,28^ Therefore, PD-L1 mutation and amplification status were evaluated in 15 patients with archived tissue using WES, and PD-L1 amplification was found in 11/15 patients. However, consistent with previous literature, PD-L1 amplification did not always correlate with high positive PD-L1 expression in IHC analysis.^29^ In this study, 6 out of 7 (85.7%) of the HL patients, 1 NK/T lymphoma patient, 1 melanoma and 1 thymoma patient showed high levels of PD-L1 expression in IHC analysis.

Our study focused on assessing TMB, but its impact on pediatric tumors was limited. Prior research has suggested that roughly 5% of sporadic pediatric solid tumors fall into the category of hypermutant. Grobner et al. reported that mutation frequencies in pediatric cancer types were 14 times lower overall than those in adult cancers.^30^ Another study found that the majority of PD-L1-amplified tumors (84.8%) had a low to intermediate TMB.^29^ In our study, all tumors had low TMB, except for one patient with choroid plexus papillary carcinoma who had an intermediate TMB of 8.16 mut/Mb as determined by high-throughput sequencing with 295 cancer gene panels.

Despite the promising activity observed in lymphoma, the ORR appears to be lower in solid tumors, as previous reported in other studies. In our study, we did not find any indications for single-agent activity of sintilimab in non-lymphoma pediatric solid tumors. However, some pediatric tumor histologies, including thymoma and choroid plexus papillary carcinoma, showed a response to PD-1 treatment and demonstrated a durable clinical benefit. We utilized germline mutations identified through WES to predict the response to ICIs. Our findings revealed previously unreported CDC27 and FGFR1 mutations in thymoma. CDC27 was found to be positively correlated with PD-L1 in T-LBL,^31^ and FGFR1 was shown to promote tumor immune evasion via the YAP/PD-L1 regulatory axis.^32^ In our cohort, the HL patient with ERBB4, MAP3K9, NF1, and SMG1 mutations, despite exhibiting high PD-L1 expression and amplification, as well as TIL positivity, achieved SD with a short treatment duration. This outcome may be attributed to the ERBB4 mutation, as ERBB family mutations have been associated with poor response to PD-1 inhibitors in NSCLC,^33^ although the underlying mechanism remains undisclosed. Overall, pediatric tumors have a low mutation burden, and certain gene mutations in crucial functional pathways may serve as predictors of the response to ICIs. Future studies are warranted to determine whether children with specific gene pathway mutations would benefit from PD-1 inhibitors.

In conclusion, our study demonstrates the tolerability of sintilimab in children. Although single-agent efficacy was not evident in most of the solid tumors in this study, we observed anti-tumor activity in HL. Future research should explore the use of sintilimab or other PD-1/PD-L1 inhibitors, either in monotherapy or as part of combination therapies with other drugs. This exploration should focus on specific pediatric patient populations with solid tumors identified through biomarkers indicating increased tumor immunogenicity.

## Materials and methods

### Design and participants

This was a multicenter, phase I, open-label, dose-escalation and dose-expansion study that aimed to evaluate the safety, tolerability and anti-tumor activity of sintilimab as a single agent in pediatric patients with advanced cancers refractory to standard therapy. Sintilimab was administered to all patients via intravenous (IV) infusion once every 3 weeks (Q3W). The study protocol received approval from the review boards of the Sun Yat-sen University Cancer Center and Children’s Hospital of Chongqing Medical University. A data safety monitoring board oversaw the study, which was conducted in accordance with the Declaration of Helsinki and international standards of good clinical practice.

To be eligible for the study, all participants were required to meet certain criteria. These included having an adequate performance status, which was assessed using the ECOG score ranging from 0 to 1. In addition, patients needed to have satisfactory hematological function, with an absolute neutrophil count of at least 1000 cells per μL, platelet count of at least 100,000 per μL, and hemoglobin level of at least 9 g/dL for all patients. Renal function was also assessed, with a creatinine clearance of ≤1.5 times the upper limit of normal for the patient’s age. Hepatic function was evaluated based on the total bilirubin level, which had to be ≤2.5 times the upper limit of normal for the patient’s age. Furthermore, patients were required to have a minimum of 42 days elapsed since their last autologous stem cell transplant, stem cell infusion, or cellular therapy before they were eligible for enrollment in the study. Initially, patients with allogenic stem cell transplants were not included. There were certain exclusion criteria as well. Patients with active brain metastases, individuals requiring daily systemic corticosteroids, those who had received systemic corticosteroids within 7 days prior to the study, and individuals who had previously undergone treatment with an anti-PD-1, anti-PD-L1, or anti-CTLA-4 medication were deemed ineligible for participation in the study. Before enrollment, all patients or their legal guardians were required to provide written informed consent. The study protocol underwent comprehensive review and received approval from the central institutional review board.

The dose escalation phase, referred to as Part A, followed a traditional ‘3 + 3’ design. The intended dose groups for Part A were 1 mg/kg, 3 mg/kg, and 10 mg/kg administered Q3W. Each dose level enrolled a minimum of three patients, who received a single IV infusion of sintilimab over 30-60 minutes. These patients were closely monitored for any signs of toxicity during a 28-day observation period. The dose escalation process followed a predefined protocol: if no DLTs occurred in the initial three patients at a particular dose level, the trial progressed to the next higher dose cohort. If one DLT emerged in the initial 3 patients, an additional 3 patients were enrolled, totaling six. If only one of these 6 patients experienced a DLT, dose escalation continued. However, if two or more DLTs occurred in a cohort, the previous lower dose level was established as the maximum tolerated dose (MTD), and further escalation was halted. Enrollment could extend to up to 12 patients in Part B, which focused on additional safety and efficacy assessments. During Part B, patients were administered IV infusions of sintilimab at doses of 1, 3, or 10 mg/kg Q3W. Radiologic evaluations were performed until indications of disease progression, intolerable adverse effects, or voluntary study withdrawal. The latest survival follow-up data was available as of December 15, 2022. The study is no longer ongoing and is closed to new participants. The primary endpoints involved identifying DLTs at the highest administered dose, evaluating safety and tolerability, while the secondary endpoints included assessing the proportion of patients with objective responses, PFS and OS.

Within 28 days following the initial administration of the investigational drug, a DLT was characterized by the occurrence of specific events associated with sintilimab. These events included grade 4 hematological toxicity lasting for over 7 days, grade 3 thrombocytopenia with bleeding tendency or requiring platelet transfusion, grade 3 neutropenia fever with bacteremia or sepsis, grade 3 pneumonia, other grade 3 or 4 non-hematological toxicity excluding grade 3 arthralgia/myalgia/myositis, grade 3 asthenia/fatigue, manageable grade 3 vomiting, grade 3 or 4 liver transaminase elevation lasting <7 days, manageable grade 3 hypertention).

### Tumor biopsies and immunohistochemistry

Prior to the administration of sintilimab, tumor biopsies were collected from the subjects. These biopsies could be either archival samples or freshly obtained. Immunohistochemistry (IHC) staining was conducted on formalin-fixed paraffin-embedded (FFPE) tumor tissue samples to identify the presence of PD-L1 (using DACO, 22C3) and CD8 + T cells (using Abcam, C8/144B, ab17147). The IHC staining process adhered to the recommended protocols provided by the respective manufacturers. Two certified pathologists evaluated PD-L1 expression on tumor cells by examining stained tissue samples.

Regarding the 22C3 antibody, PD-L1 expression was assessed using two scoring systems: the Combined Positive Score (CPS) and the TPS. The CPS was calculated by taking the number of PD-L1-stained cells, which includes tumor cells, lymphocytes, and macrophages, and dividing it by the total count of viable tumor cells. The resulting quotient was then multiplied by 100. The TPS, however, represented the proportion of viable tumor cells exhibiting partial or complete membrane staining at any intensity. The calculation methodology for CPS and TPS can be found in www.agilent.com/cs/library/usermanuals/public/29219_pd-l1-ihc-22C3-pharmdxgastric-interpretation-manual_us.pdf. A cutoff score of ≥1 for CPS and ≥1% for TPS indicated a positive result. Within the CPS system, only intratumoral and peritumoral immune cells located within a single 20× field from the tumor nest edge were considered for scoring, while stromal immune cells situated away from the tumor were excluded from the analysis.

### Genomic profiling and assessment of tumor mutational burden

We conducted comprehensive genomic profiling using WES on paired samples from 15 patients, including FFPE tissue and peripheral blood specimens. Genomic DNA was extracted from FFPE slides containing a minimum of 20% tumor content and fragmented to approximately 250 base pairs using sonication. A DNA library was then generated using the KAPA Hyper Prep Kit from KAPA Biosystems. The capture of exonic regions was performed using the Agilent SureSelect approach, following the manufacturer’s instructions (Agilent, Santa Clara, CA). Paired-end sequencing was carried out on the HiSeq2500 next-generation sequencing instrument from Illumina (San Diego, CA), resulting in 100-base reads from each end of the DNA fragments. Various genomic alterations, including single base substitutions (SNVs), short and long insertions/deletions (INDELs), CNVs, and gene rearrangements and fusions, were analyzed. The TMB was assessed by analyzing somatic mutations to estimate the overall mutational load in the tumor samples.

### Cytokine detection

The Human 27-Plex Luminex assay for cytokines was performed on serum samples of 10 patients taken pretreatment and post-treatment after 2 cycles of sintilimab. All serum samples from patients were stored at −80 °C without repetitive freezing and thawing. Cytokine levels were determined using the Luminex liquid suspension chip detection method by Wayen Biotechnologies (Shanghai, China).

### Statistical analysis

PFS and OS were evaluated utilizing the Kaplan-Meier method, and differences between the curves were assessed using the log-rank test. Statistical significance was determined with a two-tailed P-value of less than 0.05. All analyses were conducted using SPSS statistical software version 22 (IBM Corp) and Prism version 8.0 (GraphPad).

### Supplementary information

Supplementary Materials for Safety and clinical efficacy of sintilimab (anti-PD-1) in pediatric patients with advanced or recurrent malignancies in a phase I study

## Data Availability

The datasets created and/or analyzed in the course of this study can be obtained from the corresponding author upon a reasonable request.
