# Supplementary material for: Safety and clinical efficacy of sintilimab (anti-PD-1) in pediatric patients with advanced or recurrent malignancies in a phase I study
Source: Signal Transduct Target Ther. 2023 Oct 13;8:392. doi: 10.1038/s41392-023-01636-9 (PMC10570390; doi:10.1038/s41392-023-01636-9)
Supplement: Supplementary file 1 — Supplementary Materials for Safety and clinical efficacy of sintilimab (anti-PD-1) in pediatric patients with advanced or recurrent malignancies in a phase I study [file 41392_2023_1636_MOESM1_ESM.docx]

Supplementary Materials for

Safety and clinical efficacy of sintilimab (anti-PD-1) in pediatric patients with advanced or recurrent malignancies in a phase I study

Yi Que^1#^, Juan Wang^1#^, Feifei Sun^1#^, Shan Wang^2#^, Jia Zhu^1^, Junting Huang^1^, Zhenzhen Zhao^2^, Li Zhang^1^, Juan Liu^1^, Jiaqian Xu^1^, Zijun Zhen^1^, Xiaofei Sun^1^, Suying Lu^1*^, Yizhuo Zhang^1*^

Correspondence to:

Suying Lu([lusy@sysucc.org.cn](mailto:lusy@sysucc.org.cn)), Yizhuo Zhang([zhangyzh@sysucc.org.cn](mailto:zhangyzh@sysucc.org.cn));

**This PDF file includes:**

Supplementary Text

Figures. S1 to S7

Table S1

**Supplementary Text**

Inclusion and exclusion criteria

**Inclusion Criteria:**

1. Age: 1-18 years old.

2. ECOG PS score: 0-1.

3. Pediatric malignant tumors confirmed by histopathology, including Hodgkin's lymphoma, mediastinal large B-cell lymphoma, NK/T-cell lymphoma, nasopharyngeal carcinoma, malignant melanoma, neuroblastoma, hepatoblastoma, sarcoma, brain tumor, etc.

4. Advanced patients who have not responded to standard treatment.

5. Presence of at least one measurable lesion.

6. Estimated survival time of at least 6 months.

7. Heart function:

a. LVEF ≥ 50% as assessed by color Doppler echocardiography.

b. No myocardial ischemia detected by EKG.

c. No history of arrhythmia requiring drug intervention prior to admission.

8. Patients must have fully recovered from the acute toxicity of all previous anticancer chemotherapy.

a. For myelosuppression chemotherapy: at least 21 days after the last myelosuppression chemotherapy (42 days if nitrosourea was used in an earlier stage).

b. For experimental drug or non-chemotherapy anticancer therapy: must not have been used within 28 days prior to the planned start of sintilimab use, and must have fully recovered from any clinically significant therapy-related toxicity.

c. For immunotherapy: at least 42 days after completion of any type of immunotherapy (excluding steroids), including immune checkpoint inhibitors and tumor vaccines.

d. For X-ray therapy (XRT): at least 14 days after local palliative XRT (small oral area); for other significant bone marrow (BM) irradiation, including pre-radioiodinated m-iodobenzidine (131I-MIBG) treatment, at least 42 days must have elapsed.

e. For stem cell infusion without total body irradiation (TBI): no evidence of active graft-versus-host disease, and at least 56 days must have passed since transplantation or stem cell infusion.

9. Patients who have previously received CTLA-4 antibody must meet the following conditions to be eligible:

a) More than 12 weeks since the last administration.

b) No history of serious immune-related adverse events (CTCAE V4.03 G3 or G4).

10. For patients with known absence of bone marrow (BM) involvement:

a. Neutrophil absolute count (ANC) ≥ 1 × 109/L.

b. Platelet count ≥ 100 × 109/L.

c. Hemoglobin ≥ 90 g/L.

11. Liver and kidney function should meet the following criteria:

a. Bilirubin (combined + unconjugated) ≤ 2.5 times the upper limit of normal value (ULN) (age-adjusted); patients with confirmed Gilbert's syndrome can be assessed at the researchers' discretion.

b. Aspartate aminotransferase (AST) and alanine aminotransferase (ALT) ≤ 2.5 times ULN.

c. Estimated glomerular filtration rate (eGFR) ≥ 30 ml/min/1.73 m2 or serum creatinine (CR) ≤ 1.5 times ULN.

12. Ability to comply with outpatient treatment, laboratory monitoring, and necessary clinical visits during the study.

13. Availability of parents/guardians to understand, agree, and sign the study's informed consent form (ICF) and the applicable child consent form before initiating any relevant procedures. The subjects themselves must have the capacity to express their consent (when applicable), with the consent of the parents/guardians.

**Exclusion Criteria:**

1. Received treatment with anti-PD-1 or anti-PD-L1 monoclonal antibody or related pathway targeted drugs.

2. Known allergy to PD-1 monoclonal antibody or any of its adjuvants, or a history of allergic diseases or severe allergic constitution.

3. Patients with other malignant tumor diseases, except for those treated by the institute, unless they have been cured with no recurrence within 3 years prior to study selection, have completely removed basal cell and squamous cell skin cancer, or have completely removed any type of carcinoma in situ.

4. Active central nervous system metastasis (treated or untreated), including symptomatic brain metastasis, meningeal metastasis, or spinal cord compression, etc. Exceptions: asymptomatic brain metastasis (no progression within at least 4 weeks after radiotherapy and/or no neurological symptoms or signs after surgical resection, with no need for dexamethasone or mannitol treatment).

5. Uncontrolled pleural effusion, pericardial effusion, or ascites requiring repeated drainage.

6. Persistence of toxicity from previous treatment higher than grade 1 (CTCAE V4.03 Standard), except for hair loss and neurotoxicity.

7. History of mental disorders.

8. History of drug use or drug abuse upon inquiry.

9. History of idiopathic pulmonary fibrosis or pneumonia.

10. Complications requiring treatment with immunosuppressive drugs or systemic/local corticosteroids at immunosuppressive doses (prednisone > 10 mg/day or equivalent dose of similar drugs).

11. History of autoimmune diseases, including but not limited to systemic lupus erythematosus, psoriasis, rheumatoid arthritis, inflammatory bowel disease, Hashimoto's thyroiditis, etc. Exceptions: type I diabetes, controllable hypothyroidism with hormone replacement therapy, skin diseases not requiring systemic treatment (such as vitiligo, psoriasis), controlled celiac disease, or diseases that do not recur without external stimulus.

12. Active or previous TB infection.

13. Active infection requiring systemic treatment.

14. Uncontrolled hypertension (systolic ≥ 140 mmHg and/or diastolic ≥ 90 mmHg), pulmonary hypertension, or unstable angina pectoris; myocardial infarction, bypass surgery, or stent placement within 6 months prior to administration; history of NYHA class 3-4 chronic heart failure; clinically significant valvular disease; severe arrhythmia requiring treatment (excluding atrial fibrillation and paroxysmal supraventricular tachycardia); QTc interval ≥ 450 ms in males or ≥ 470 ms in females (calculated using Fridericia's formula); cerebrovascular accident (CVA) or transient ischemic attack (TIA) within 6 months prior to administration, etc.

15. Serious medical conditions, including but not limited to uncontrolled diabetes, active peptic ulcer, active bleeding, etc.

16. Positive results for anti-HIV, TP AB, and HCV AB; positive HBV AG and HBV DNA copy number higher than the upper limit of normal value.

17. Abnormal thyroid function (FT3, FT4, T3, T4).

18. Major surgery expected within 28 days before administration or during treatment.

19. Planned administration of live or attenuated vaccines within 4 weeks before administration, during treatment, or within 5 months after the last administration.

20. Participation in another clinical trial and receipt of the investigational drug treatment within 30 days before administration.

21. Patients considered unsuitable for the trial based on the investigator's judgment due to other reasons.

**Fig S1:**


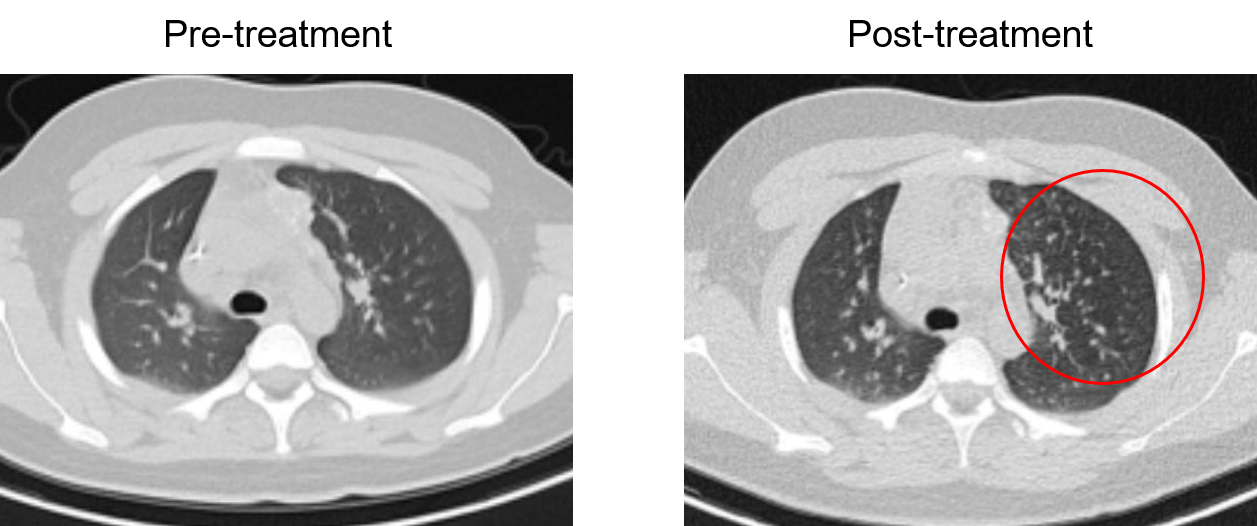


**Fig S1.**  A patient with primary mediastinal large B-cell lymphoma developed Grade 1 immune pneumonia after receiving 5 courses of sintilimab treatment.

**Fig S2**


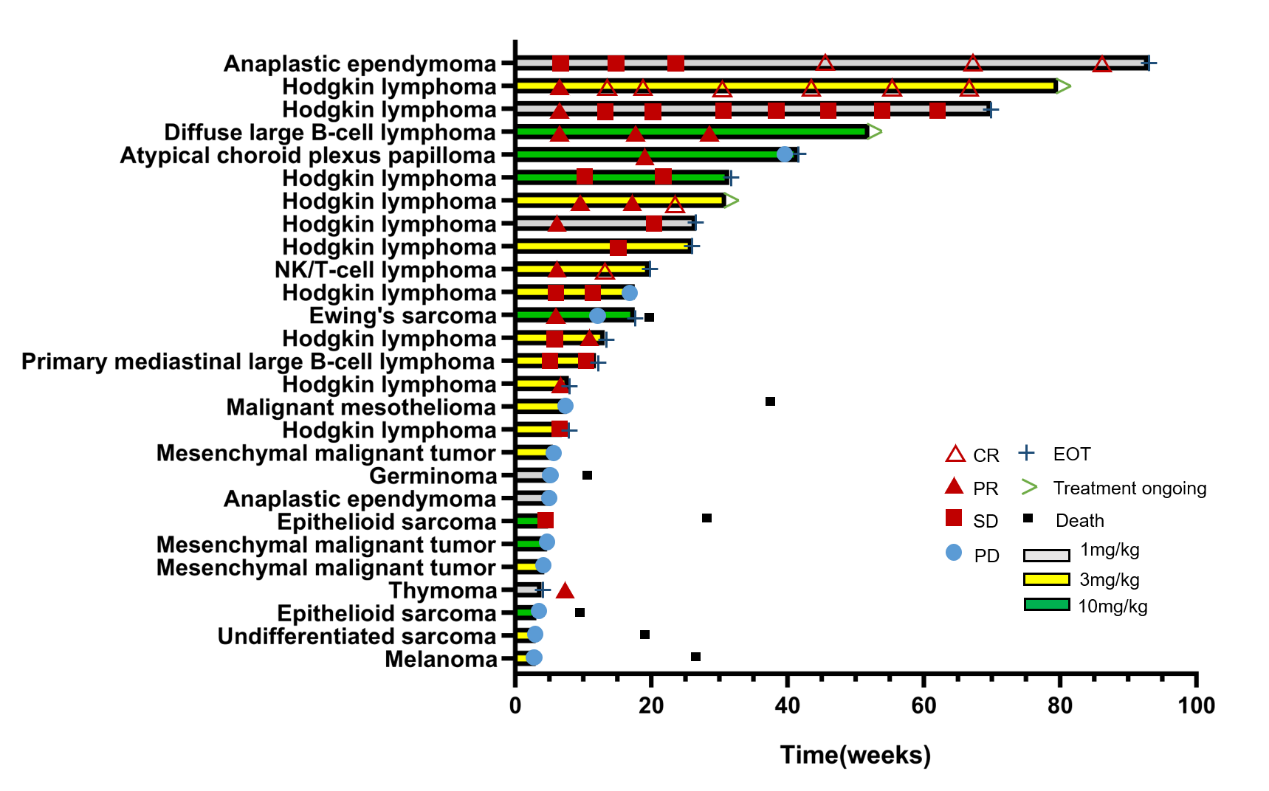


**Fig.S2** Response of sintilimab. Each bar represents one patient. The length of each bar represents the duration of treatment of each patient.

**Fig S3**


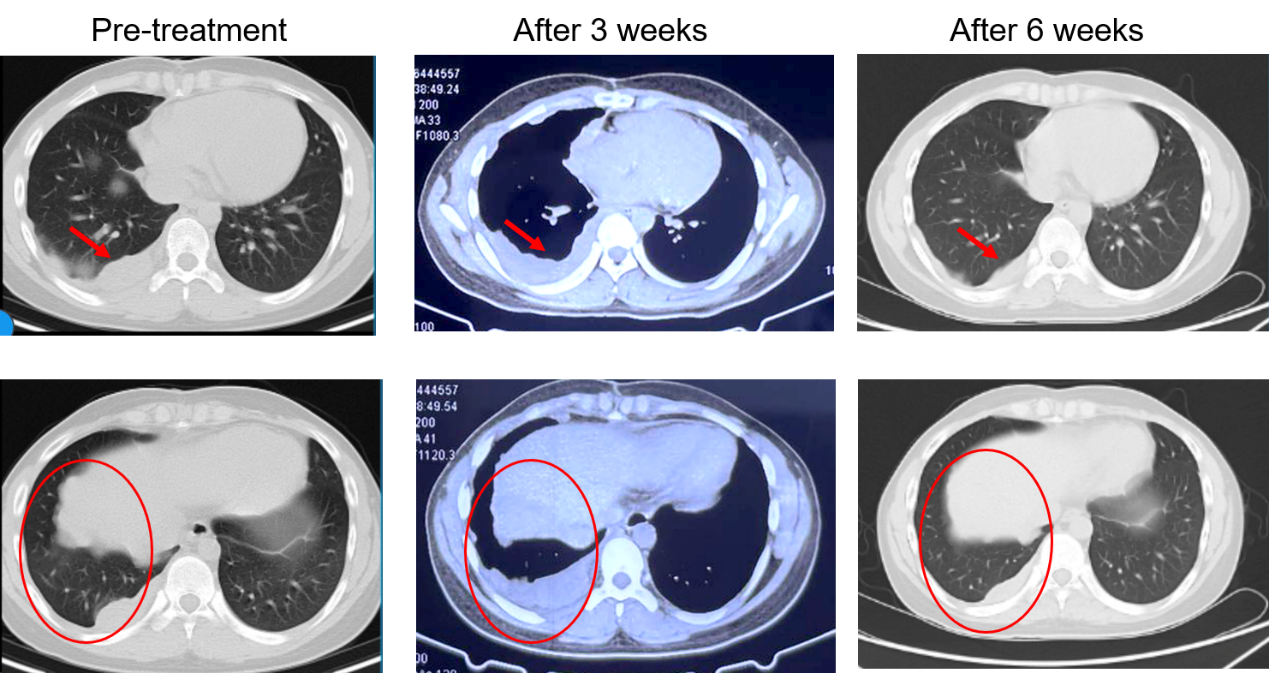


**Fig. S3** Imaging features of a patient with thymoma following treatment with sintilimab were observed. After 3 weeks of treatment, the tumor showed signs of enlargement, leading to treatment discontinuation due to toxicity. However, after 6 weeks, the tumor gradually started to shrink and achieved a PR.

**Fig S4**


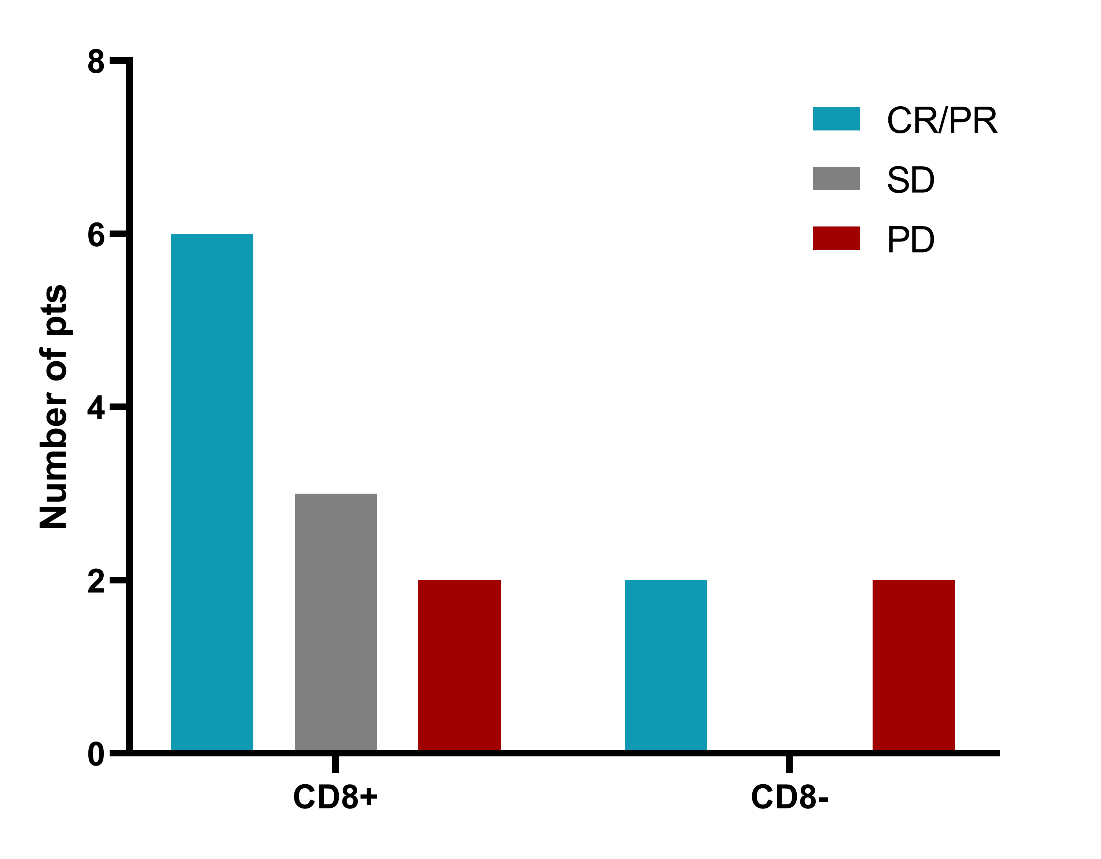


**Fig. S4** CD8 positive (n=11) and CD8 negative(n=4) expression subgroups determined by IHC staining on infiltrating lymphocytes are compared for clinical response.

**Fig S5.**


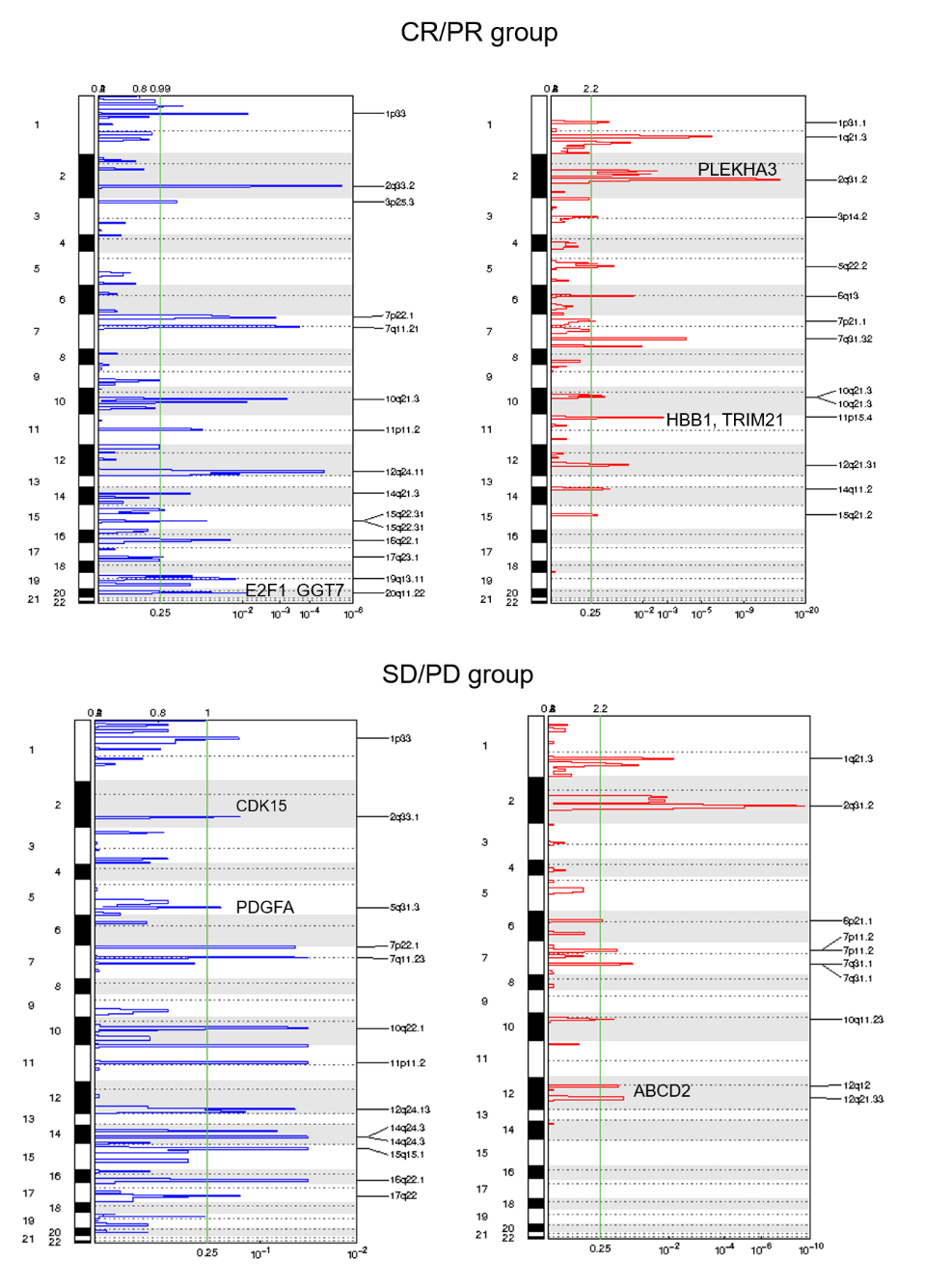


**Fig. S5** Copy number analysis of the response group (PR/CR) and the non-response group (PD/SD).

**Fig S6**


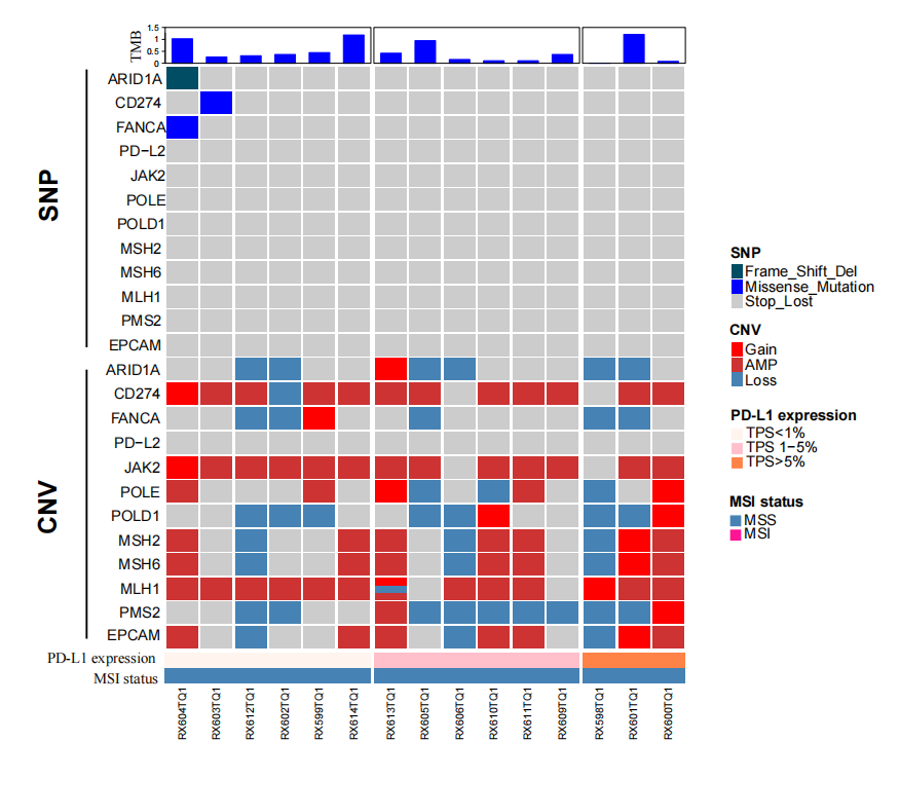


**Fig. S6** Bars showing TMB (top), heatmaps summarizing alterations of MSH related genes and predicted MSI status (bottom) treated with sintilimab.

**Fig S7.**


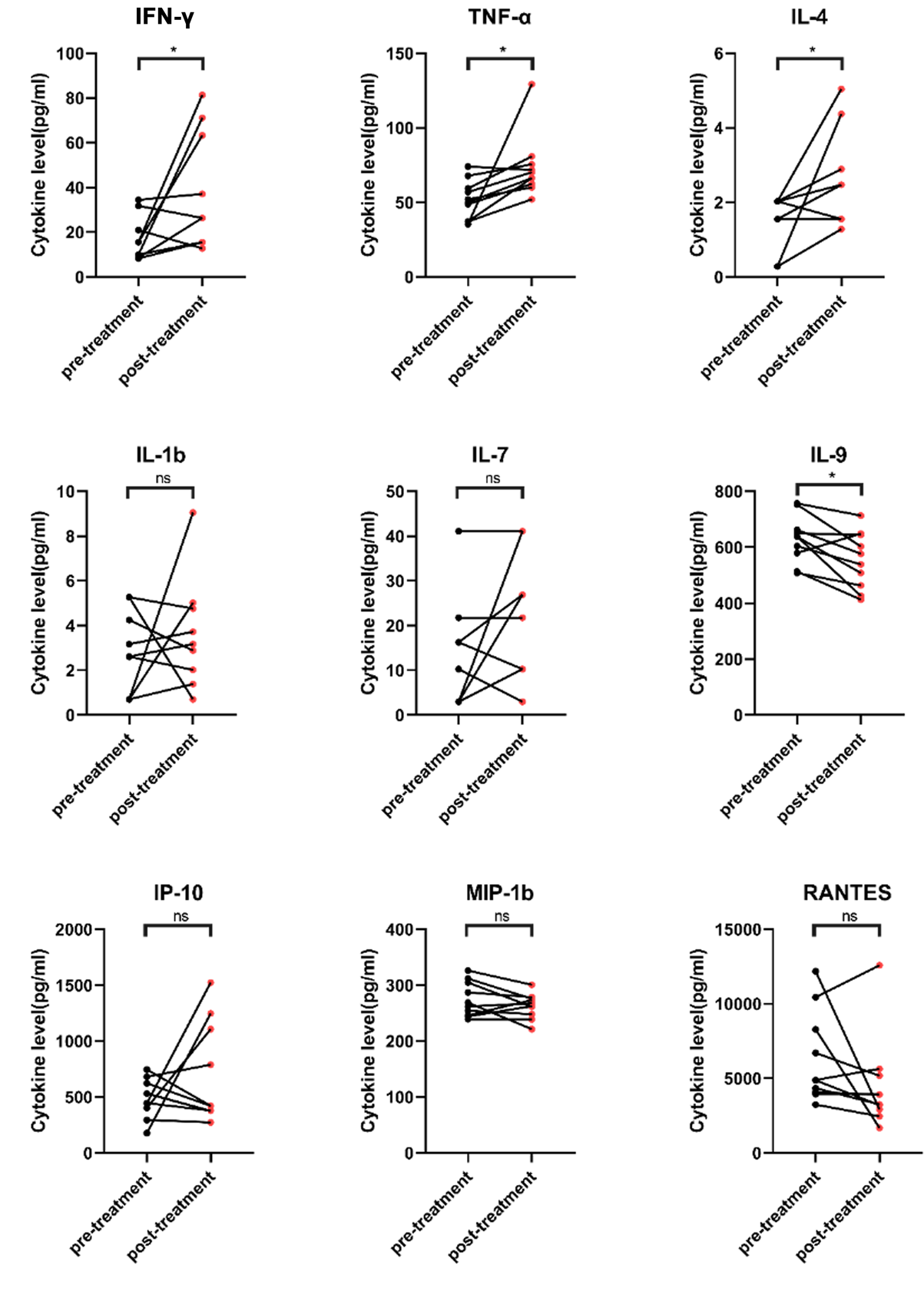


**Fig. S7** Pre- and post-treatment plasma levels of IFN-γ, TNF-α, IL-4, IL-9, IL-1b, IL-7, IP-10, MIP-1b and RANTES in 10 patients in the combination treatment group. Paired t-test, **p* < 0.05. Error bars indicate mean± SD.

**Table S1 Summary of sintilimab safety profile for hematological tumors and solid tumors**

|  | Hematological tumors(n=13) | Solid tumors(n=16) | Hematological tumors(n=13) | Solid tumors(n=16) |
| --- | --- | --- | --- | --- |
|  | Grade 1-2 | Grade 1-2 | Grade 3-4 | Grade 3-4 |
| Total number of patients | 13(100%) | 13(81.3%) | 1(7.7%) | 2(12.5%) |
| Anaemia | 5(38.5%) | 5(31.3%) | 0 | 0 |
| Fatigue | 1(7.7%) | 1(6.3%） | 0 | 0 |
| Pyrexia | 7(53.8%) | 4(25%) | 0 | 0 |
| Aspartate aminotransferase increased | 2(15.4%) | 0 | 0 | 1(6.3%) |
| Alanine aminotransferase increased | 2(15.4%) | 0 | 0 | 1(6.3%) |
| Hypothyroidism | 2(15.4%) | 3(18.8%) | 0 | 0 |
| Hyperthyroidism | 2(15.4%) | 2(12.5%) | 0 | 0 |
| Nausea | 0 | 1(6.3%） | 0 | 0 |
| Rash, maculopapular | 2(15.4%) | 1(6.3%） | 0 | 0 |
| Diarrhoea | 1(7.7%) | 0 | 0 | 0 |
| Abdominal pain | 2(15.4%) | 1(6.3%） | 0 | 0 |
| Decreased white blood cell count | 3(23.1%) | 1(6.3%） | 0 | 0 |
| Decreased appetite | 0 | 2(12.5%) | 0 | 0 |
| Pruritus | 4(30.8%) | 0 | 0 | 0 |
| Decreased platelet count | 1(7.7%) | 1(6.3%） | 1(7.7%) | 1(6.3%) |
| Arthralgia | 0 | 1(6.3%） | 0 | 0 |
| Decreased neutrophil count | 3(23.1%) | 0 | 0 | 0 |
| Pneumonitis | 1(7.7%) | 0 | 0 | 0 |
| Hypoalbuminemia | 0 | 2(12.5%) | 0 | 0 |
| Hypokalaemia | 1(7.7%) | 0 | 0 | 0 |
| Hypocalcaemia | 0 | 1(6.3%） | 0 | 0 |
| Cough | 0 | 3(18.8%) | 0 | 0 |
| Myalgia | 1(7.7%) | 1(6.3%） | 0 | 0 |
| Headache | 2(15.4%) | 1(6.3%） | 0 | 0 |
| Vomitting | 0 | 2(12.5%) | 0 | 0 |
| Chest tightness | 1(7.7%) | 1(6.3%） | 0 | 0 |
| Constipation | 0 | 2(12.5%) | 0 | 0 |
| Myositis | 0 | 0 | 0 | 1(6.3%) |
